# Behind the Bait: Delving into *PhishTank's* hidden data

**DOI:** 10.1016/j.dib.2023.109959

**Published:** 2023-12-15

**Authors:** Affan Yasin, Rubia Fatima, Javed Ali Khan, Wasif Afzal

**Affiliations:** a School of Software, Northwestern Polytechnical University, Xian 710072, Shaanxi, China; b School of Software, Tsinghua University, Beijing, China; c Department of Computer Science, School of Physics, Engineering & Computer Science, University of Hertfordshire, Hatfield, UK; d School of Innovation, Design and Engineering, Mälardalen University, Västerås, Sweden

**Keywords:** Phished URL, Social engineering, Email security, Web security, Computer security, Artificial intelligence, Dataset

## Abstract

Phishing constitutes a form of social engineering that aims to deceive individuals through email communication. Extensive prior research has underscored phishing as one of the most commonly employed attack vectors for infiltrating organizational networks. A prevalent method involves misleading the target by employing phishing URLs concealed through hyperlink strategies. *PhishTank*, a website employing the concept of crowd-sourcing, aggregates phishing URLs and subsequently verifies their authenticity. In the course of this study, we leveraged a Python script to extract data from the *PhishTank* website, amassing a comprehensive dataset comprising over 190,0000 phishing URLs. This dataset is a valuable resource that can be harnessed by both researchers and practitioners for enhancing phish- ing filters, fortifying firewalls, security education, and refining training and testing models, among other applications.

## Data Specification

For enhanced clarity, the data specifications are presented in Table 1.

**Table 1:** Specification Table.

Sr.No	Specification	Explanation
1.	*Subject*	Software Engineering, Computer Science
2.	*Specific Subject Area*	Information Security, Artificial Intelligence
3.	*Data Format*	Raw csv file
4.	*Type of Data*	Table
5.	*Data Collection How data was acquired*	Data were extracted from publicly available lists of phishing and legitimate URL on the phishtank website.
6.	*Data Source Collection*	The data was collected from two geographical locations e.g, Pakistan and China.
7.	*Code file*	python file (.py) for replication and extension.
8.	Smaller dataset accessibility	1.Repository name: Mendeley Data 2.Data identification number: 10.17632/8py4n46nby.1 3.Direct URL to data: https://data.mendeley.com/datasets/8py4n46nby/1
9.	Bigger dataset accessibility	1.Repository name: Zenodo Data 2.Data identification number: 10.5281/zenodo.8371046 3.Direct URL to data: https://doi.org/10.5281/zenodo.8371046
10.	PhishTank website used for (Phished or Legitimate) URL retrieval	https://phishtank.org/phish_archive.php

## 1 Value of Data

• Over 190,0000 URLs have been extracted, providing an extensive dataset for re- searchers and practitioners to utilize.
• The dataset encompasses instances of phishing URL obtained from *PhishTank*^1^. This data is suitable for utilization within various machine learning processes, including model training and prediction, among others.
• The dataset exhibits versatility and can serve various purposes, including:
  - Training classifiers for identifying phishing URLs.
  - Analyzing URL patterns.
  - Serving as an evaluation benchmark.
  - Facilitating the development of browser plugins or email filters.
  - Training reinforcement learning agents.
  - Examining trends over time.
  - Enhancing phishing education efforts.
• Machine learning and data mining researchers, as well as information security professionals, can derive significant value from these datasets. The data serves as a valuable resource for developing firewall solutions, intelligent ad-blocking mechanisms, and systems for the detection of malware.
  Below mentioned are several ways in which the phishing URL dataset could contribute to the enhancement of firewall solutions against phishing attacks:
  - Generating URL blocklists.
  - Enhancing URL pattern recognition.
  - Facilitating the development of learning models.
  - Assessing the firewall's performance.
• These datasets can be employed for *educational purposes* in the classroom to :
  - instruct on the differentiation between phishing and legitimate URLs.
  - conduct user-centered analysis on the extracted Phished URLs.
  - Creating phishing awareness games and simulations.
  - Engaging in class activities to practice identifying and analyzing phishing URLs.
• The dataset is suitable for constructing classification models and can serve as a performance benchmark for the development of cutting-edge machine learning techniques. Below mentioned is one approach to benchmark novel phishing URL detection tech- niques against established methods using this dataset:
  - Split the dataset into training, validation, and test sets.
  - Implement established phishing URL detection methods like blacklists, URL rule- based classifiers, existing ML models as baselines. Train and optimize them on the training set.
  - Develop new phishing URL detection techniques - this could be new engineered features, different ML algorithms like DNNs, ensemble models, etc. Train the novel models on the training set.
  - Evaluate the performance of both the baseline and new models on the common hold-out test set. Metrics like accuracy, precision, recall, F1-score, ROC curve can be used.
  - The benchmarks allow directly comparing how the novel techniques stack up against existing standard approaches. The metrics quantify the gains achieved by the new methods.
  - Analyze the errors made by both old and new techniques to understand why they succeed or fail in certain cases. This provides insights into limitations and areas for improvement.
  - The benchmarks help assess which novel phishing URL detection techniques are promising and which need further refinement before real-world deployment.
  - The standardized benchmark and test set facilitates rapid iteration between developing new models and evaluating their performance against multiple baselines using a common dataset.
• Moreover, the dataset holds potential for various applications, including benchmarking novel phishing URL detection techniques against established methods, conducting research to identify innovative features for distinguishing phishing URLs through in- depth analysis of the examples, developing browser plugins or email filters for phishing URL detection, creating visualization tools to emphasize distinctions between phishing and legitimate URLs, and establishing public blacklists or blocklists containing verified phishing URLs based on the gathered samples.
• For enhanced clarity, the data specifications are presented in Table 1.

## 2 Data Description

• The dataset is contained in CSV files named in the format *Valid Phishes Offline (n).xlsx*. The small glimpse is presented in the Table 2. Additionally, a comprehensive explanation of each attribute within these files is provided below to facilitate reader comprehension:
  - ***ID -*** The unique identifier assigned to the submitted URL on PhishTank.
  - ***Phish URL -*** Link to view the phishing URL report on PhishTank website. URL can be further explored on the phishtank website by visiting the given link in the column.
  - ***Phish -*** The actual submitted URL submitted on the website.
  - ***Submitted (Information) -***Data and Time when the URL was submitted to PhishTank.
  - ***Submitted User Link -*** The web-link to the Phishtank user who submitted the phishing information.
  - ***Valid Phished -*** Label indicating this URL has been verified as phishing by PhishTank.
  - ***Online/Offline -*** Indicates if the phishing URL is still active or offline at the time of data collection.

**Table 2:** Dataset attributes.

ID	Phish URL	Phish	Submitted Date	Submitted user link	Valid	Online /Offline
2293803	https://phishtank.org/phish detail.php?phish id=2293803	http://www.atelier-capelli.pl/images/cielo.com.br/Ourocard/	added on Feb 19th 2014 4:49 AM	https://phishtank.org/user.php?username=ZRSABUSE	VALID PHISH	Offline
2293802	https://phishtank.org/phish detail.php?phish id=2293802	http://gest.pl//images/smilies/Bradesco/	added on Feb 19th 2014 4:49 AM	https://phishtank.org/user.php?username=demartin	VALID PHISH	Offline
2293800	https://phishtank.org/phish detail.php?phish id=2293800	http://continentalvirtual.6te.net/core/cielo/copa2014/promocao/premiad	added on Feb 19th 2014 4:49 AM	https://phishtank.org/user.php?username=demartin	VALID PHISH	Offline
2293798	https://phishtank.org/phish detail.php?phish id=2293798	http://paypal.com.login.account.id.user.346679784563466784.webscr-md5-	added on Feb 19th 2014 4:48 AM	https://phishtank.org/user.php?username=PhishReporter	VALID PHISH	Offline
2293787	https://phishtank.org/phish detail.php?phish id=2293787	http://www.productosblackberry.com/images/paypal.com/View.php?#/ flow&	added on Feb 19th 2014 4:20 AM	https://phishtank.org/user.php?username=cleanmx	VALID PHISH	Offline
2293786	https://phishtank.org/phish detail.php?phish id=2293786	http://www.productosblackberry.com/images/paypal.com/Suite.php	added on Feb 19th 2014 4:20 AM	https://phishtank.org/user.php?username=cleanmx	VALID PHISH	Offline

## 3 Experimental Design, Materials and Methods

Social engineers [1] manipulate individuals working for the organization by disseminating infected links, files, or malware (phishing attacks [2,3]). Through these deceptive tactics, the unwitting human assets inadvertently grant social engineers unauthorized access to the system.

In the course of compiling the phishing URL datasets, we employed a Python script to retrieve phishing URL data from the *PhishTank* website 2. The extraction procedure is explicated in the paper as an pseudo-code and algorithm. Throughout this procedure, we obtained a comprehensive phishing URL dataset comprising over 190,0000 entries. To enhance readability and evaluation, the acquired list was subsequently annotated. The culmination of this effort resulted in the creation of three CSV files, each containing extracted features [4]. These CSV files are convenient and compatible with various tools and programming libraries, facilitating ease of use and analysis.

The following steps elucidate the process for regenerating data from the provided script or code file located within the designated folder.

1. Begin by installing the most recent version of Python.
2. Access the PhishTank website and utilize the filters 3 provided on the site for conduct- ing searches.
3. Copy the updated website link and paste it into the Python code provided, as illus- trated in the following example.

driver.get(‘https://phishtank.com/phish archive.php’)

1. Establish a new directory bearing the name “phishtank”.
2. Position the file named “phishtank.py” within the aforementioned “phishtank” folder.
  • Generate a new CSV file.
  • Name the newly created file as “Data.csv.”
3. Execute the program or script in the Command Prompt (Cmd).

**Code Snippet:** The subsequent code snippet is employed for extracting data from the *PhishTank* website.



## Limitation

One limitation of this study pertains to the character length of reported phishing URLs within PhishTank. While the study imposed a limit of 70 characters, certain URLs listed on PhishTank exceeded this threshold. Consequently, only the initial 70 characters of such lengthy URLs were captured in the dataset. Nevertheless, it is worth noting that even with this limitation, the retrieved URLs provide a substantial amount of pertinent information. For instance, the URL provided as an example^4^ exceeds 70 characters in length; however, due to the imposed constraint, only the initial 70 characters were extracted in the URL.

## Ethics Statement

This study confirms that the current work does not involve human subjects, animal experiments, or any data collected from social media platforms thus did not require any approval.

## CRediT authorship contribution statement

**Affan Yasin:** Conceptualization, Methodology, Writing – original draft, Writing – review & editing. **Rubia Fatima:** Conceptualization, Methodology, Writing – original draft. **Javed Ali Khan:** Software, Data curation, Visualization, Investigation, Writing – review & editing. **Wasif Afzal:** Software, Validation, Formal analysis, Writing – review & editing, Funding acquisition.

## Data Availability

PhishTank URL's (Original data) (Zenodo) A Collection of Phished URLs AND Code (Original data) (Mendeley Data)

## Footnotes

1 https://phishtank.org.

3 https://phishtank.org/phish archive.php.

## Footnotes

2 https://phishtank.org.

4 http://www.buysellleasetrade.com/m1/webmail administrator password res.
